# Stressful life events among incarcerated women and men: Association with depression, loneliness, hopelessness, and suicidality

**DOI:** 10.1186/s40352-021-00140-y

**Published:** 2021-08-24

**Authors:** Kelly E. Moore, Shania Siebert, Garrett Brown, Julia Felton, Jennifer E. Johnson

**Affiliations:** 1 Department of Psychology, East Tennessee State University, 420 Rogers-Stout Hall, P.O. Box 70649, Johnson City, TN 37614 USA; 2 College of Human Medicine, Michigan State University, Flint, USA

**Keywords:** Stressful life events, Depression, Suicide, Perceived social support, Prison, Incarceration, Gender

## Abstract

**Background:**

Justice-involved populations report a higher than average number of pre-incarceration stressful life events. However, few studies have described stressful life events which occur during incarceration, explored gender differences in these events, or evaluated the effect of these events on well-being.

**Method:**

This study draws from a sample of male and female adults incarcerated in 6 prison facilities across two states (*n* = 160) to identify the number and type of stressful life events they experienced during incarceration, gender differences in stressful events, and the relationship between stressful life events and markers of well-being (i.e., depression, hopelessness, loneliness, suicidality). We also examined whether perceived social support would buffer the relationship between stressful events and well-being outcomes.

**Results:**

Participants on average reported experiencing 4 stressful life events during their current incarceration, the most common being relocation to another cell and being made fun of/insulted by someone in the prison. There were few gender differences in types of events experienced. Regression analyses showed that stressful life events were associated with more loneliness, as well as suicidality, but only when participants had low perceived social support.

**Conclusions:**

Stressful life events, and drawing on social support networks to cope with stress, should be addressed in the context of correctional treatments to reduce suicide risk during incarceration.

## Introduction

Justice-involved individuals comprise a sizeable portion of the U.S. population, with almost 11 million adults entering the criminal justice system each year (Zheng, 2020) and 1 in 38 under some form of correctional supervision (i.e., detained in jail or prison, on probation or parole; Kaeble & Cowhig, 2018) at any given time. Justice-involved individuals, more so than the general population, are chronically exposed to stressful interpersonal, financial, legal, and other life events (Harlow, 2003; Radatz & Wright, 2017; McDaniels-Wilson & Belknap, 2008; Scott, Lurigio, Dennis, & Funk, 2016). In general, stressful life events are thought to have a serious negative impact on well-being (Banyard, Williams, & Siegel, 2003; Fowler, Allen, Oldham, & Frueh, 2013; Hopfinger, Berking, Bockting, & Ebert, 2016), including among justice-involved populations (Gunter, Chibnall, Antoniak, McCormick, & Black, 2012; Messina & Grella, 2006; Radatz & Wright, 2017; Scott et al., 2016). In particular, stressful events that have happened throughout childhood and adulthood are consistently related to depressed mood (Ahmad & Mazlan, 2014; Keaveny & Zauszniewski, 1999; Turney, Wildeman, & Schnittker, 2012), low social support (Asberg & Renk, 2014; Kane & Dibartolo, 2002; Kao et al., 2014; Singer, Bussey, Song, & Lunghofer, 1995), and suicidality (Fazel, Ramesh, & Hawton, 2017; Gunter et al., 2012; Jones & Maynard, 2013) among people in the criminal justice system.

Justice-involved individuals are not only repeatedly exposed to stressors while in the community, but they also experience stressful events while incarcerated, when they have even less access to social or financial resources that could help them cope. In fact, incarceration prompts a variety of negative, stressful experiences (Carlson & Shafer, 2010; Fogel, 1993; Kupers, 1996). Stressors such as having a romantic relationship end, being assaulted, or losing custody of a child, as well as stressors specific to the incarceration context such as being denied parole or having issues with other incarcerated people or staff, are rarely emphasized in studies of stress, despite their potential impact on adjustment (Blaauw, Winkel, & Kerkhof, 2001; Buchman-Schmitt et al., 2017). Given that incarceration presents an opportunity to access and treat chronically stressed and vulnerable people prior to their re-entry into the community, it is important to understand the types of stressful events that occur during incarceration and how they ultimately impact well-being.

### Stressful life events during incarceration

According to stress process theory, if a person’s trajectory of life events is consistently moving toward their goals and values and is viewed to be acceptable within society, that person should have a stable life course with minimal trauma and overall good health (Pearlin, Schieman, Fazio, & Meersman, 2005). However, for many individuals who are justice-involved, childhood and adulthood stressors beget behavior contrary to societal norms, which begets additional stressors (Maschi, Viola, & Morgen, 2014). While many studies have demonstrated that incarcerated individuals report a wide variety of stressful events throughout the course of their lives, including physical or sexual abuse, violence, unsuccessful court decisions, separation from children, termination of partner relationships, financial difficulties, difficulty finding employment, homelessness, and life-threatening accidents among other events (Carlson & Schafer, 2010; Gosein, Stiffler, Frascoia, & Ford, 2016; Maschi, Morgen, Zgoba, Courtney, & Ristow, 2011; Messina & Grella, 2006), fewer studies have examined stressful events that occur during periods of incarceration.

Incarceration involves significant exposure to stress. Theorized by Sykes (1958) as the “pains of imprisonment,” incarceration involves a loss of liberty, desirable goods and services, intimate relationships, autonomy, and security, all of which cause stress and impact well-being. These “pains” refer to the ways in which physical confinement places strains on existing relationships with spouses, children, and other sources of social support as well as remove opportunities to form new relationships that are critical for coping with stress. They also refer to incarcerated people’s lack of choice in who they interact with, what situations they are in, and how they live their lives, all within an environment that can involve high rates of violence. Qualitative studies with formerly and currently incarcerated people show that interpersonal interactions that are unique to the prison environment, including feeling mistreated, harassed, or judged negatively by correctional officers and medical staff, as well as fear of violence and negative interactions with other incarcerated people, are the most commonly reported stressors during incarceration (Maschi, Viola, & Koskinen, 2015; Porter, 2019). Other  interpersonal stressors are often reported as well, including social isolation, lack of visitation or calls from family/friends, and infidelity or the ending of romantic relationships (Vanhooren, Leijssen, & Dezutter, 2017). Qualitative studies suggest that incarceration is particularly stressful for women, showing that separation from family and children, loss of freedom and autonomy, and lack of treatment options have a significant impact on women’s well-being and adjustment (Douglas, Plugge, & Fitzpatrick, 2009; Fogel, 1993; Harner & Riley, 2013).

There have been fewer quantitative studies on stress which occurs during incarceration. One study showed that 50% of people incarcerated in jail experienced life stressors (i.e., divorce, financial problems, serious mental or physical illness, emotional and physical neglect, separation from children, and sexual assault) while incarcerated, the most commonly reported being serious mental or physical illness, forced distance from children, and sexual harassment and abuse (Gosein et al., 2016). In an earlier study, Hochsteler and colleagues (2004) examined potentially traumatic events that occurred in prison among 208 males who had recently been released. On average, participants indicated being victimized (i.e., by theft, scams, robbery, property destruction, threats, assault) once per month during incarceration (*SD* = 2.63). These studies, however, did not capture a wide range of stressors, including those that are unique to the incarceration context, and had primarily male samples. Most quantitative studies to date have examined lifetime exposure to stressors with incarcerated men or women separately, and have shown that both genders report experiencing similar numbers of stressful events, but not necessarily the same types of events (Carlson & Schafer, 2010; Gunter et al., 2012). There may be gender differences in the types of stressful life events people experience *during incarceration*, given that the incarceration environment often differs greatly for women and men.

### Stressful life events and well-being

In general, incarcerated individuals who have experienced more life stressors (not specifically focused on stressors during incarceration) are more likely to be diagnosed with mental health conditions including PTSD (Gosein et al., 2016), mood disorders (Ahmad & Mazlan, 2014; Fogel, 1993; Gunter et al., 2012; Keaveny & Zauszniewski, 1999; Wanklyn, Day, Hart, & Girard, 2012), and anxiety disorders (Gunter et al., 2012; Keaveny & Zauszniewski, 1999). Depression in particular has been underscored as a problematic consequence of life stress, and is correlated with the number of life stressors incarcerated individuals experience (Ahmad & Mazlan, 2014; Fogel, 1993; Hurley & Dunne, 1991; Keaveny & Zauszniewski, 1999; Senol-Durak & Gencoz, 2010; Turney et al., 2012). Studies show that experiencing stress (not necessarily during incarceration) is also correlated with suicidal ideation and risk (Blaauw, Arensman, Kraaij, Winkel, & Bout, 2002; Chapman, Specht, & Cellucci, 2005; Gunter et al., 2012) as well as near-death suicide attempts (Rivlin, Hawton, Marzano, & Fazel, 2013) among incarcerated individuals. Stressful life events have also been found to be associated with factors that contribute to depression and suicidality, such as hopelessness and loneliness among incarcerated individuals (Asberg & Renk, 2014; Biggam & Power, 1997; Kao et al., 2014; Merten, Bishop, & Williams, 2012; Wanklyn et al., 2012). Moreover, overall trauma experiences are related to lower perceived social support among people who are incarcerated (Kao et al., 2014).

The stress experienced during incarceration can be particularly detrimental to well-being. Incarceration stress can disrupt the standard sequence of “normal” life events (e.g., marriage, having children, school, entering the work force) and cause additional issues on top of pre-incarceration challenges (Pearlin, 2010). Some studies find an association between perceived stress (Ahmad & Mazlan, 2014; Fogel, 1993; Senol-Durak & Gencoz, 2010) as well as the anticipation of stressful events that could happen in the incarceration context (i.e., unfavorable court decisions, serious arguments or altercations with prisoners or staff, other difficulties; Fogel, 1993; Hurley & Dunne, 1991) with depression symptoms during incarceration. Other studies indicate that events such as being bullied by another incarcerated person are correlated with suicide risk (Blaauw et al., 2001). However, research in this area is greatly lacking, and the degree to which stressors experienced during incarceration have negative impacts on well-being is unknown. Further, it is important to examine the impact of incarceration stress on well-being among samples of incarcerated men and women, given the lack of women represented in quantitative studies and the well-established gender differences in stress and coping (Matud, 2004).

### Factors that buffer the impact of stressful life events

Perceived social support may play a role in the relationship between stressful life events and well-being during incarceration, especially given that incarcerated individuals typically have very limited access to social support systems. Studies among the general population show the relationship between life stressors and depression is stronger among people who have low perceived social support (Cheong, Sinnott, Dahly, & Kearney, 2017) and that social support can buffer the effects of negative life events on outcomes such as suicide (Kleiman, Riskind, & Schaefer, 2014). Social support is thought to be a critical protective factor among justice-involved individuals, in that emotional and material support “lessens the effects of exposure to criminogenic strains” (Cullen, 1994). Likewise, lack of social support is thought to exacerbate the stressors that justice-involved people experience and contribute to maladjustment (Cullen, 1994). Studies among justice-involved individuals show that social support is linked to better mental health and adjustment, buffering the impact of depression on substance use (Mowen, Boman IV, & Schweitzer, 2020) and exposure to stressors on engagement in violent/criminal behavior (Maschi, 2006; Robbers, 2004). Support during incarceration in particular (e.g., visitation, phone calls) is linked to better connectedness with family and better mental health (Folk, Stuewig, Mashek, Tangney, & Grossmann, 2019). Lack of perceived social support may be a risk factor that exacerbates the association between stressful life events and poor well-being among incarcerated individuals.

### Current study

The present study utilizes a sample of incarcerated women and men to: (1) describe stressful life events experienced during incarceration; (2) compare number and kinds of stressful life events men and women experience during incarceration; (3) examine the association between stressful live events during incarceration and markers of well-being (e.g., self-reported depression, clinician-rated depression, hopelessness, loneliness, suicidality); and (4) investigate whether perceived social support moderates the relationship between stressful events and poor well-being.

## Methods

### Participants and procedures

Participants were women and men between the ages of 18 and 65 who were part of a randomized controlled trial (RCT) that was designed to test the effectiveness of interpersonal therapy (IPT) for major depressive disorder (MDD) among people incarcerated in prison (Johnson et al., 2019). A total of 181 prisoners from six prisons across Rhode Island and Massachusetts were randomized into group IPT with treatment as usual (TAU) or to TAU alone. The jail and prison systems in these states are combined, and the facilities included in this study were medium security men’s facilities and women’s facilities that housed women of all security levels. The data used in the current study were collected at the baseline assessment after participants enrolled; any currently incarcerated persons were eligible regardless of the amount of time served on their sentence. For the present study, people incarcerated for less than 6 months were excluded since questions referred to stressors that occurred in the past 6 months and we wanted to assess stress that occurred during incarceration, resulting in a final sample of 160. Participants had to meet Diagnostic and Statistical Manual of Mental Disorders IV criteria for current primary (non-substance induced) MDD and report that they expected to stay at their current facilities for at least 6 months. People who met the lifetime criteria for bipolar disorder or a primary psychotic disorder, who were in prison residential mental health treatment, or could not understand English were excluded. Research assistants with a BA or MA administered structured interviews and self-report measures at the prisons. All research assistants were trained in interviewer administered instruments at Brown University’s Clinical Assessment and Training Unit and were supervised.

### Measures

#### Sociodemographics

Participant demographics (i.e., gender [man = 1, woman = 0], race/ethnicity [white = 1, racial/ethnic minority = 0], age, marital status [never married = 1, other = 0], number of prior offenses, and number of months served on the current sentence) were collected via self-report.

#### Stressful life events

Stressful life events were assessed using a modified version of the Life Events Questionnaire (LEQ; Brugha & Cragg, 1990). The original LEQ is a 12 item self-report measure that asks  people to report whether or not they have experienced stressful life events in the past 6 months. Some of the original items on the LEQ such as; “Experienced a serious illness or injury?”, “Experienced the death of a close friend or family member?”, “Gotten divorced or legally separated from a spouse?”, and “Had something valuable lost or stolen?” were retained, whereas others were dropped because they applied to all incarcerated individuals (i.e., recent unemployment, legal problems). In addition, 22 items were added based on stressful events that incarcerated people often experience and can have a positive or negative impact on mood, some of which were unique to the incarceration context. Example items include; “Lost custody of a child permanently or had parental rights terminated,” “Anniversary of a sad or traumatic event,” “Put in isolation,” “Serious financial difficulties,” and “New charges or convictions.” Items were rated as either 0 (did not happen) or 1 (did happen in the past 6 months). If the item is rated as 1, participants are asked to give the date of the event. For the current study, to remain consistent with research focused on *negative* life events, 6 stressful events that were judged as likely to have a positive impact on mood were excluded from the scale creation (i.e., gotten back together with a spouse or partner, resolved a problem with a family member/close friend, learned you may regain custody of a child permanently, made parole, gotten a prison job, started exercising more). The 26 other events were added to calculate the total number of stressful life events participants experienced in the past 6 months. Studies have found the original LEQ to be reliable and valid among prison samples (Brugha & Cragg, 1990). Of our 26 items, the item “denied parole” decreased the scale alpha and was thus dropped, resulting in a 25 item scale. In the present sample, the Kuder-Richardson 20 (KR-20) measure of internal consistency for scales with dichotomous choices was .63, which is considered acceptable (Davey, Gugiu, & Coryn, 2010). In addition to the total LEQ, we selected the 9 stressful events that were unique to incarceration contexts to create a separate scale (i.e., had a serious problem with another incarcerated person, had an argument with prison staff, made fun of/insulted by someone in the prison, put in isolation, had a disciplinary action taken against you, had problems with prison discharge plans, new charges or convictions, loss of good time, denied parole); being denied parole contributed to a low alpha again and was dropped. The resulting 8 item scale of incarceration-specific stressors had an alpha of .64 on the KR-20 index.

#### Depressive symptoms

Depressive symptoms were measured using two scales which captured unique elements of cognitive, affective, and somatic components of depression severity and which were assessed by both self-report and clinical judgment. The Hamilton Rating Scale for Depression (HRSD, Hamilton, 1960) is a 17-item interview measure administered by a clinician or researcher that assesses symptoms of depression experienced over the past week. Some of the symptoms assessed using the HRSD include; insomnia, guilt, helplessness, and lack of reactivity. The scoring on the Hamilton varies based on symptom, with 8 of the items being scored 0–4 and 9 of the items being scored 0–2. Scores are added to get the total depression score. Higher total scores indicate more severe depression symptoms (Hamilton, 1960). The Quick Inventory of Depressive Symptomatology (QIDS; Rush et al., 2003) is a 16 item self-report measure used to assess the severity of depressive symptoms. The measure asks individuals to rate common depressive symptoms on a scale of 0 (*asymptomatic*) -3 (*severe*) and includes items such as; “Feeling sad”, “Sleeping too  much”, and “Feeling  restless.” The total score is calculated by adding scores from the following criteria; sad mood (item 5), concentration (item 10), outlook on self (item 11), suicidal ideation (item 12), general interest (item 13), energy or fatigue (item 14), the highest score on any of the four sleep items (items 1–4), the highest score on any of the appetite or weight change items (items 6–9), and the highest score on the two psychomotor items (items 15–16). The total range for the final score is 0–27 with a higher score meaning more severe depressive symptomology (Rush et al., 2003).

#### Hopelessness

Hopelessness was measured using the Beck Hopelessness Scale (BHS, Beck, Weissman, Lester, & Trexler, 1974), a 20 item self-report measure that was designed to measure three different aspects of hopelessness: feelings about the future, loss of motivation, and future expectations. The measure evaluates individuals’ feelings of optimism/pessimism about the future and includes items such as; “Things just won’t work out the way I want them to” and “I look forward to the future with hope and enthusiasm.” Items are rated as true or false and the total “hopelessness score” is calculated by summing the individual items. The BHS demonstrated good reliability in this study (alpha = .93).

#### Suicidality

Suicidality was measured using the Beck Scale for Suicidal Ideation (BSI, Beck, Kovacs, & Weissman, 1979), a 21 item self-report measure that assesses past week suicidal ideation (i.e., wishes to die, desire to make an active or passive suicide attempt, duration of ideation, and frequency of ideation), behaviors, and plans. Items 1 through 19 use a 3-point scale, ranging from 0 to 2, to assess the severity of the participants suicidal thoughts, as well as their characteristics and the participant’s attitude about them. Individual scores are added together to produce the total BSI score (range 0–38). Items 20 and 21 assess whether or not participants have had any previous suicide attempts and are not included in the total BSI score. The BSI demonstrated good reliability in this sample (alpha = .87).

#### Loneliness

Loneliness was measured using the UCLA Loneliness Scale (UCLA LS; Russell, 1996). The original UCLA Loneliness Scale is a 20-item self-report scale designed to measure one’s subjective feelings of loneliness and feelings of social isolation. A few items include; “How often do you feel left out?”, “How often do you feel that there is no one you can turn to?”, and “How often do you feel outgoing and friendly?” We used a 10-item version due to time constraints. Responses are rated on the following scale: “I never feel this way “(1), “I rarely feel this way” (2),” I sometimes feel this way” (3), and “I often feel this way” (4) and summed to create a total score. Higher scores indicate higher feelings of loneliness. The UCLA LS demonstrated good reliability in this sample (alpha = .85).

#### Perceived social support

Perceived social support was assessed using the 12-item self-report Multidimensional Scale of Perceived Social Support (MSPSS, Zimet, Dahlem, Zimet, & Farley, 1988), which assesses perceived support from family, friends, and significant others. Items capture whether participants believe they have people who care about them, whether they can talk about their problems with others, and whether they have people to help them in times of need. Responses are rated on a Likert Scale from 1, very strongly disagree, to 7, very strongly agree and summed to provide a total score. The MSPSS demonstrated excellent reliability in this sample (alpha = .93).

### Data analysis

Data were analyzed using SPSS version 26. Descriptive statistics and bivariate correlations were run to describe the LEQ and study variables. Gender differences in sociodemographics, the number of stressful life events, and each type of stressful life event were examined with independent samples t-tests and chi-square tests. Within the SPSS PROCESS macro, the LEQ and perceived social support were entered as predictors of self-reported and clinician-reported depression symptoms, hopelessness, suicidality, and loneliness in separate linear regression models to understand main effects on outcomes. The interaction between the mean centered LEQ and perceived social support was included on a second step of each model. This approach was repeated for models including the 8-item incarceration-specific stressful events predictor. Interactions significant at *p* < .05 were graphed using PROCESS-generated code in order to demonstrate the relationship between stressful life events and well-being outcomes at low, medium, and high levels of social support (i.e., 1 SD below the mean, the mean, and 1 SD above the mean). Sociodemographics significantly associated with outcomes at the bivariate level were included as covariates in all models.

## Results

### Sample descriptors

Participants were 160 adults (70% male) aged 18–65 years old (*M* = 40 years, *SD* = 10.3) who were incarcerated in prison in Rhode Island (65.4%) or Massachusetts (34.6%). A total of 62.5% identified as white, 23.8% identified as Black, 1.3% identified as Asian, 6.3% identified as Native American or Alaskan Native, and 12.5% identified as other race; 81.8% identified as non-Hispanic. Most participants had never been married (58.5%) and self-reported an average of 12.7 prior arrests (SD = 17.4) Participants had been incarcerated for a range of 6 to 488 months (*M* = 78, *SD* = 88.6) at the time of the baseline interview, with men having been incarcerated longer (*M* = 97 months, *SD* = 98.0) than women (*M* = 34 months, *SD* = 32.4), *t* (152) = − 6.1, *p* < .001).

### Stressful life events during incarceration

Participants reported experiencing between 0 and 13 stressful life events (*M* = 3.9, *SD* = 2.7) during the past 6 months. The most common stressful life event participants reported experiencing was having been moved to another cell (38.6%), followed by having been made fun of by someone in the prison (34.8%), having had disciplinary action taken against them by prison staff (32.3%) and the anniversary of a sad or traumatic event (32.3%), and major financial difficulties (31.6%). The least common stressor was receiving new charges or convictions (0.6%).

#### Gender differences

Chi square analyses examining stressful life events by gender are displayed in Table 1 with significant differences bolded. There was no difference in the overall number of stressful life events reported for men and women. Women were more likely than men to report having a child in trouble and men were more likely than women to report having gotten into a fight.

**Table. 1:** Number/percentage of stressful life events endorsed in the past 6 months at study baseline by gender

	Women	Men	*χ* ^2^ or *t*
**Stressful Life Events**			
Number of stressful life events (mean)	3.5	4.0	−1.0
Any stressful life event	87.5%	91.8%	0.7
Serious illness or injury	2.1%	6.4%	1.3
Serious illness or injury of a close relative	22.9%	23.6%	0.0
Death of an immediate family member	8.5%	10.9%	0.2
Death of a friend/extended family member	19.1%	16.4%	0.2
Divorced/legally separated from spouse/partner	4.2%	0.9%	1.9
Had a romantic relationship end	14.6%	12.7%	0.1
Had a serious problem with a spouse/partner	18.8%	19.1%	0.0
Had a serious problem with a close friend/other relative	18.8%	20.0%	0.3
Had major financial difficulties	27.1%	33.6%	0.7
Had something valuable lost or stolen	4.2%	6.4%	0.3
Learned you may lose custody of a child permanently	2.1%	1.8%	0.0
Lost custody of a child permanently/parental rights terminated	4.2%	1.8%	0.7
Been assaulted	2.1%	1.8%	0.0
**Got into a fight**	**0.0%**	**9.1%**	4.7*
**Had a child in trouble**	**16.7%**	**5.5%**	5.2*
Anniversary of a sad or traumatic event	22.9%	36.4%	2.8
Had a serious problem with another inmate	8.3%	19.1%	2.9
Denied parole	4.2%	12.7%	2.7
Had an argument with prison staff	16.7%	28.2%	2.4
Had disciplinary action by the prison taken against you	31.3%	32.7%	0.0
Put in isolation	12.5%	22.7%	2.2
Made fun of/insulted by someone w/in the prison	35.4%	34.5%	0.0
Been moved (i.e., to a different cell)	45.8%	35.5%	1.5
Had problems with prison discharge plans	0.0%	5.5%	2.7
New charges or convictions	0.0%	0.9%	0.4
Loss of good time	16.7%	15.5%	0.0

*p < .05

### Stressful life events and well-being

Descriptive statistics and bivariate correlations are displayed in Table 2. At the bivariate level, stressful events in the past 6 months were associated with more clinician-rated depression, loneliness, hopelessness, and suicidality. Correlation patterns were consistent for stress specific to the incarceration context. Gender and prison location were related to poorer well-being on several measures, thus warranting inclusion as covariates in all models. Results of regression models examining stressful life events on outcomes are displayed in Table 3, and those examining incarceration-specific stressful life events on outcomes are displayed in Table 4. The variance inflation factor was examined and ranged from 1.0 to 1.2 for all regression models, which is well below problematic levels and indicates that multicollinearity did not influence our results. Across all models examining stressful life events and incarceration-specific stressful events as predictors of well-being outcomes, there was a significant negative main effect of social support. Stressful life events and incarceration-specific stressful events were both significantly related to loneliness. In addition, incarceration-specific stressful events were also significantly related to self-reported depression severity (see Tables 3 and 4).

**Table. 2:** Descriptive statistics and bivariate correlations among primary study variables

	N (%)	M (SD)	Actual Range	2.	3.	4.	5.	6.	7.	8.	9.	10.	11.	12.	13.	14.
Variable																
1. Gender (man)	112 (70.0%)		0–1	.13	−.09	.03	.03	.33***	.08	.11	−.26**	.10	.06	.23**	.19*	.12
2. Age		40.0 (10.3)	20–61	–	.16*	−.46***	.24**	.25**	−.18*	−.18*	−.04	−.09	−.07	−.02	.09	.08
3. Race (white)	100 (62.5%)		0–1		–	−.20*	.20*	−.11	−.06	−.01	−.05	−.06	.07	.01	.10	.13
4. Never married	94 (58.8%)					–	−.20*	.04	.15	.23**	.08	.03	−.02	−.00	.00	−.00
5. Prior arrests		12.7 (17.4)	0–100				–	−.09	−.04	−.05	−.25**	−.01	.04	.11	.15	.16
6. Months incarcerated		78.0 (88.6)	0–488.5					–	−.19*	−.18*	.08	−.15	−.07	−.12	−.07	.03
7. Stressful events		3.9 (2.7)	0–13						–	.80***	−.10	.18*	.15	.26**	.16*	.23**
8. Incarceration-specific stressful events		1.5 (1.6)	0–8							–	−.06	.17*	.12	.34***	.18*	.19*
9. Perceived social support		51.8 (18.6)	12–84								–	−.27**	−.25**	−.47***	−.48***	−.39***
10. Depression (HRSD)		25.6 (7.2)	3–27									–	.62***	.38***	.49***	.36***
11. Depression (QIDS)		13.1 (4.1)	11–43										–	.35***	.47***	.38***
12. Hopelessness		8.0 (5.9)	0–20											–	.41***	.29***
13. Loneliness		29.6 (6.1)	15–40												–	.48***
14. Suicidality		3.0 (4.5)	0–21													–

*p < .05, **p < .01, ***p < .001

**Table. 3:** Multiple linear regression examining the association of stressful life events, social support, and their interaction on well-being during incarceration

	*B (SE)*	*t*	*p*	*Model R*^*2*^*/R*^*2*^*change*
DV: Depression (HRSD)				.12
Prison location	1.20 (.49)	2.48	.014	
Gender (man)	−2.38 (1.68)	−1.42	.157	
Stressful life events	.36 (.21)	1.74	.085	
Social support	−.09 (.03)	−2.92	.004	
Stress x Social support	−.00 (.01)	−.07	.941	.00
DV: Depression (QIDS)				.11
Prison location	.52 (.28)	1.86	.065	
Gender (man)	−1.20 (.96)	−1.25	.214	
Stressful life events	.14 (.12)	1.16	.249	
Social support	−.06 (.02)	−3.29	.001	
Stress x Social support	−.01 (.01)	−1.37	.172	.01
DV: Hopelessness				.29
Prison location	1.15 (.36)	3.19	.002	
Gender (man)	−1.72 (1.24)	−1.39	.167	
Stressful life events	.15 (.16)	.94	.348	
Social support	−.14 (.02)	−6.22	<.001	
Stress x Social support	−.02 (.01)	−1.82	.071	.02
DV: Loneliness				.27
Prison location	−.02 (.37)	−0.06	.951	
Gender (man)	1.31 (1.29)	1.02	.309	
Stressful life events	.51 (.16)	3.15	.002	
Social support	−.14 (.02)	−5.71	<.001	
Stress x Social support	.00 (.01)	0.51	.611	.00
DV: Suicidality				.29
Prison location	1.09 (.28)	3.97	<.001	
Gender (man)	−2.31 (.95)	−2.42	.017	
Stressful life events	.22 (.12)	1.87	.064	
Social support	−.10 (.02)	−5.43	<.001	
**Stress x Social support**	**−.02 (.01)**	**−2.38**	**.019**	**.03**

*HRSD* Hamilton Rating Scale for Depression, *QIDS* Quick Inventory of Depressive Symptomatology

**Table. 4:** Multiple linear regression examining the association of incarceration-specific stressful life events, social support, and their interaction on well-being during incarceration

	*B*	*t*	*p*	*Model R*^*2*^*/R*^*2*^*change*
DV: Depression (HRSD)				.13
Prison location	1.23 (.49)	2.5	.012	
Gender (man)	−2.54 (1.68)	−1.51	.134	
Incarceration-specific stressful events	.59 (.35)	1.68	.095	
Social support	−.09 (.03)	−3.05	.003	
Stress x Social support	−.01 (.02)	−.60	.552	.00
DV: Depression (QIDS)				.10
Prison location	.50 (.28)	1.80	.075	
Gender (man)	−1.24 (.97)	−1.28	.202	
Incarceration-specific stressful events	.22 (.20)	1.10	.274	
Social support	−.06 (.02)	−3.32	.001	
Stress x Social support	−.01 (.01)	−1.02	.311	.01
DV: Hopelessness				.29
Prison location	1.07 (.36)	2.98	.003	
Gender (man)	−1.73 (1.25)	−1.39	.168	
Incarceration-specific stressful events	.45 (.26)	1.73	.085	
Social support	−.14 (.02)	−6.21	<.001	
Stress x Social support	−.01 (.01)	−1.02	.309	.01
DV: Loneliness				.31
Prison location	−.07 (.36)	−.19	.847	
Gender (man)	1.25 (1.26)	.99	.322	
Incarceration-specific stressful events	1.15 (.26)	4.37	<.001	
Social support	−.14 (.02)	−6.05	<.001	
Stress x Social support	.00 (.01)	.28	.779	.00
DV: Suicidality				.28
Prison location	1.10 (.28)	3.96	<.001	
Gender (man)	−2.44 (.97)	−2.53	.012	
Incarceration-specific stressful events	.34 (.20)	1.71	.089	
Social support	−.10 (.02)	−5.46	<.001	
**Stress x Social support**	**−.03 (.01)**	**−2.41**	**.017**	**.03**

Standardized effects presented

*HRSD* Hamilton Rating Scale for Depression, *QIDS* Quick Inventory of Depressive Symptomatology

### Social support as a moderator of stressful life events on outcomes

**Fig. 1:**
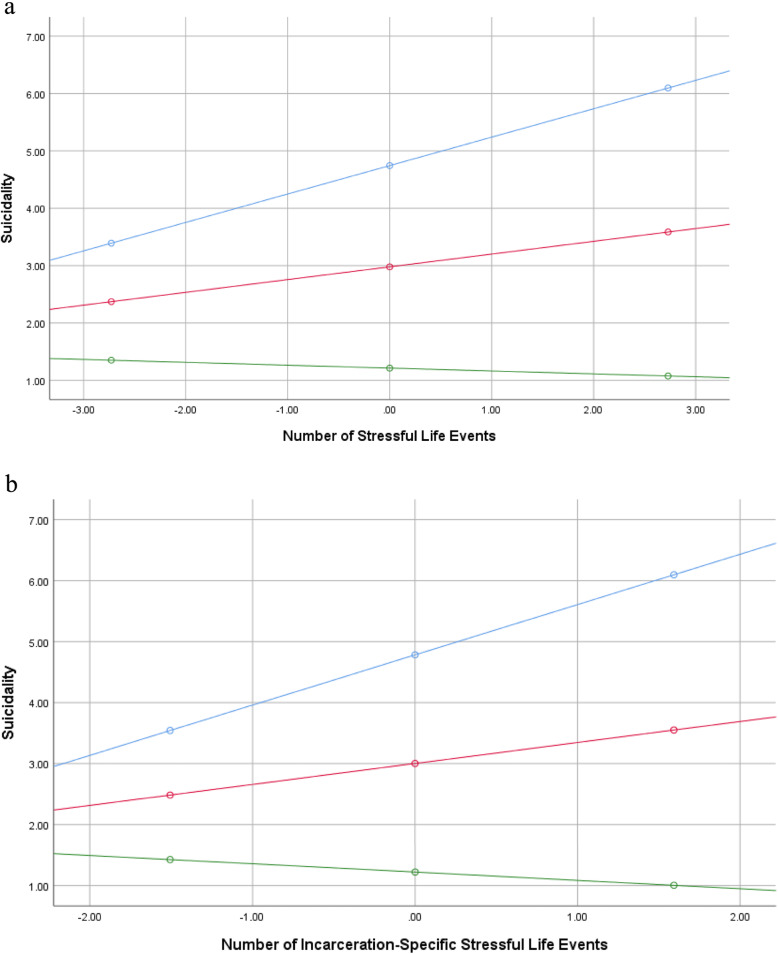
**a** The relationship between stressful life events and suicidality at low (1 SD below the mean), moderate (mean), and high (1 SD above the mean) levels of perceived social support. **b** The relationship between incarceration-specific stressful life events and suicidality at low (1 SD below the mean), moderate (mean), and high (1 SD above the mean) levels of perceived social support

There was no evidence of perceived social support moderating the relationship between stressful life events or incarceration-specific stressful events with depression symptoms, hopelessness, or loneliness, but there was a significant interaction of stressful life events (*b =* −.02, *t* (154) = − 2.38, *p =* .019) and incarceration-specific stressful events (*b =* −.03, *t* (154) = − 2.41, *p =* .017) with perceived social support in the relationship with suicidality (see Fig. 1). At low levels of social support, stressful life events (*b =* .50, *t* (154) = 3.46, *p =* .001) and incarceration-specific stressful events (*b =* −.82, *t* (154) = 3.10, *p =* .002) had a strong positive relationship with suicidality, but the relationships were nonsignificant at moderate and high levels of perceived social support.

## Discussion

Incarcerated individuals are exposed to more trauma/stressful life events than the general population (Briere, Agee, & Dietrich, 2016; Radatz & Wright, 2017), which, in turn, places them at increased risk for a number of concerning mental health outcomes, including depression and suicidality. Stressful events that occur during incarceration may be particularly important to understand. However, little is known about the frequency and impact of stressful life events during incarceration on individuals’ well-being. This study sought out to describe stressful life events experienced during incarceration and compare these events between men and women, as well as test whether more stressful life events were associated with depression, hopelessness, loneliness, and suicidality. We also tested whether perceived social support buffered the relationship between stressful events and outcomes. Our results supported some of our hypotheses.

This study found that the number of stressful life events experienced during incarceration in the past 6 months (e.g., divorce or separation, serious injury or illness, major financial difficulties, assault, being made fun of by other incarcerated people/staff, disciplinary infractions, being put in isolation, having trouble with discharge plans) was significantly associated at the bivariate level with all poor well-being outcomes with the exception of self-reported depression. These findings are consistent with other studies of incarcerated populations which show that stressful events that have happened during childhood or prior to incarceration are associated with poor well-being (Carlson & Schafer, 2010; Radatz & Wright, 2017; Rivlin et al., 2013; Wanklyn et al., 2012), and studies showing that stressful life events predict maintenance of depressive symptoms (Hurley & Dunne, 1991; Kraaij & de Wilde, 2001; Phillips, Carroll, & Der, 2015). While many of the stressful events assessed in this study were those that can happen to anyone (not just incarcerated people), they were experienced during a period of incarceration, thus potentially making them more difficult to navigate due to lack of available coping resources. Limiting our stressful events measure to incarceration-specific stressors (e.g., problems with discharge plans, being insulted or made fun of by someone in the prison) had the same pattern of results.

In regression models, experiencing more stressful life events during incarceration, including just those unique to incarceration, was linked to greater loneliness. Some of the most commonly identified stressors during incarceration are interpersonal nature, including the loss of romantic relationships and strain on family/friends, as well as feeling harassed by correctional officers and medical staff (Porter, 2019). Many of the stressful events reported by our participants were also interpersonal, including having been made fun of by someone in the prison, having serious problems with other incarcerated people, getting into fights, getting divorced, and losing custody of children. It makes sense that experiencing more of these stressors would prompt interpersonal consequences like loneliness among incarcerated people.

Our findings also show that stressful events during incarceration increase suicide risk. In multivariate models, stressful events including those just specific to incarceration marginally increase suicidality, which builds on research showing an association between trauma/stress experienced prior to incarceration and suicidality during incarceration (Blaauw et al., 2002; Messina & Grella, 2006). Importantly, this effect depended on how strong participants’ perceived social support was—a lack of perceived social support was associated with poorer well-being across all outcomes, and stressful life events (including incarceration-specific events) were only significantly associated with suicidality among people with low levels of perceived social support. These results are consistent with literature suggesting perceived social support is a protective factor against suicidality among general populations (Kleiman et al., 2014) and incarcerated individuals (Favril, Vander Laenen, Vanderviver, & Audenaert, 2017; Pratt & Foster, 2020; Richie et al., 2019). Individuals who enter incarceration with limited social support, or experience a decrease in social support during incarceration, may feel that they have no one to talk to about what they are going through or to help them navigate difficult stressors. This lack of interpersonal connection to cope with stressors may exacerbate suicidal thoughts. The prison environment is not known for being a therapeutic environment for those who are vulnerable to the negative effects of stress, such as people with serious mental illness (Gosein et al., 2016; Kupers, 1996). Stress may be particularly important to monitor among such high risk groups in order to decrease suicidal behavior while individuals are in custody. In addition, research should explore other support-related factors that may buffer the effect of stress on suicide risk during incarceration, including visitation from friends and family or engagement with correctional mental health services.

During the 6-month time period observed, men and women reported experiencing similar numbers of stressful life events, but significantly more women reported that they had a child in trouble. Studies of lifetime stressors among incarcerated parents show that women are more likely to report having a child who died than men were (Carlson & Schafer, 2010), and qualitative data highlights the unique struggles that mothers experience related to separation from children during incarceration (Douglas et al., 2009). Men were more likely to report getting into a fight, which is in line with research showing that women engage in fewer rule violations including violent infractions during incarceration (Celinska & Sung, 2014). Differences in rates of other reported stressful life events did not reach statistical significance. One reason for these discrepant findings may be the time frame that was investigated here (past 6 months vs. lifetime), or our study may not have been powered to detect small effects. Future research with larger samples could investigate gender differences in the relationship between stressful events and well-being differently, and consider examining two-way interactions of gender and social support on the relationship between stressful life events and well-being.

### Strengths, limitations, and future directions

The current paper makes an important contribution to the literature on stressful life events and well-being among incarcerated populations by drawing from a large sample of men and women and investigating multiple markers of well-being. We also investigate factors that may buffer the impact of stress on well-being, thus highlighting new points of intervention. Results should be considered in light of several limitations. First, participants were included in the current study based on experiencing current MDD, and this may have inflated the number of stressors reported. However, both of our depression outcomes were normally distributed and had sufficient variability, demonstrating that not everyone in our sample experienced the same degree of depression symptoms, and that these outcomes capture that variability in depression severity. We also believe that drawing from a sample of people with MDD may have also been a strength. Aside from substance use disorders, MDD is the most common disorder diagnosed among prisoners (Fazel & Seewald, 2012) and increases risk for negative outcomes during incarceration (Fazel, Hayes, Bartellas, Clerici, & Trestman, 2016). It is particularly relevant to the aims of this study given that stress increases risk for development and chronicity of MDD (Otte et al., 2016). Additionally, given that suicide was one of the outcomes of interest in this study, drawing from a sample of prisoners with MDD likely allowed us to observe more variance than we would have if drawing from a general prison population (Favril, Indig, Gear, & Wilhelm, 2020). We had sufficient variation in our depression outcomes which allowed us to examine whether stressful experiences could explain some of this variation in symptoms. Future studies should consider the impact of stressful life events on non-depressed incarcerated samples. Second, the cross-sectional design of the study cannot determine whether stressful life events predict changes in well-being or vice-versa. Indeed, a large literature supports that depressed individuals are at particular risk for generating stress within their own lives (Hammen, 2003). Thus, future studies would benefit from disentangling the temporal ordering of these constructs. Finally, although we focused in on negative stressful life events, it is impossible to know how incarcerated people felt about the stressful events that were included on our scale and it is possible that some (e.g., getting divorced, moving to a new cell) may have been experienced as positive.

### Clinical implications

These results suggest several implications for intervention efforts targeting the reduction of depression, suicidality, and related problems among incarcerated individuals. Findings point to the need to assess exposure to stressful life events, both at intake as well as at regular intervals throughout incarceration, to understand who may be vulnerable to poor mood outcomes, and to boost their capacity to effectively cope with stress. Importantly, incarcerated individuals who experience stressors such as difficulties adjusting to the prison context, feeling judged by or uncomfortable around other incarcerated people or staff, or have disciplinary infractions or moves between housing units, may have increased risk for suicide ideation. Increasing individuals’ perceived social support may improve their ability to cope with stress that occurs during incarceration and lead to less suicidality. Importantly, many incarcerated people, especially men, do not have strong social support systems in the community which could be drawn from to help mitigate suicide risk during incarceration through calls or visitation (Jiang & Winfree Jr, 2006; Wolff & Draine, 2004). Further, because incarcerated people report feeling judged and looked down upon by medical staff within correctional facilities (Porter, 2019), they may not feel comfortable seeking mental health services or support during incarceration. Alternative ideas for bolstering social support during incarceration are needed, perhaps through reducing stigma that deters help-seeking or increasing contact with certified peer recovery specialists who have a history of incarceration (Barrenger, Maurer, Moore, & Hong, 2020).

Both interpersonal therapies (IPT) and cognitive behavioral approaches and have been shown to improve individuals’ emotion regulation and reduce stress responses in the face of stressful life events (Bruijniks, DeRubeis, Hollon, & Huibers, 2019; Bulmash, Harkness, Stewart, & Bagby, 2009; Lipsitz & Markowitz, 2013). Of concern, however, is the lack of access within prisons to evidence-based interventions targeting these and related risk factors for mental health problems (Johnson et al., 2019; Johnson & Zlotnick, 2012). Recent findings from this research team suggest that implementing an evidence-based intervention (specifically, IPT) is feasible and effective for reducing depressive symptoms, hopelessness, posttraumatic stress disorder and for increasing MDD remission among incarcerated individuals (Authors names removed). The current study highlights the need for wider implementation of this and other approaches to reduce the considerable mental health burden faced by this population. Treatment addressing stressful life events among incarcerated individuals may not only increase their quality of life but may also contribute to reduced disciplinary problems and management issues within the prison, which would benefit correctional staff and the criminal justice system from a management perspective. Moreover, in addition to reducing risk for mental health problems during and after incarceration, targeting stressful experiences during incarceration may reduce recidivism risk. Sykes’ (1958) work on the pains of imprisonment suggests that exposure to difficult situations and stressors during incarceration can increase disdain for the law and connection with incarcerated peers, thereby ultimately increasing criminal behavior.

## Conclusions

Justice-involved men and women experience a wide range of stressful life events during incarceration, and these experiences are associated with depression, loneliness, and suicidality. Helping justice-involved individuals better manage life stress, and in particular, bolstering their social support networks, could reduce suicide risk during incarceration. Evidence-based interventions that focus on improving interpersonal relationships (e.g., IPT) may be beneficial toward this end, but should be adapted to consider the difficulty in fostering social connections between incarcerated people and their non-incarcerated friends/family.

## Data Availability

The datasets generated and/or analyzed during the current study are not publicly available because they require Department of Corrections approval.
